# Preconditioning Strategies to Enhance Neural Stem Cell-Based Therapy for Ischemic Stroke

**DOI:** 10.3390/brainsci10110893

**Published:** 2020-11-23

**Authors:** Farah Amna Othman, Suat Cheng Tan

**Affiliations:** School of Health Sciences, Health Campus, Universiti Sains Malaysia, Kubang Kerian 16150, Kelantan, Malaysia; farahamna88@gmail.com

**Keywords:** ischemic stroke, neural stem cells (NSC), preconditioning, cell transplantation

## Abstract

Transplantation of neural stem cells (NSCs) has been proposed as an alternative novel therapy to replace damaged neural circuitry after ischemic stroke onset. Nonetheless, albeit the potential of these cells for stroke therapy, many critical challenges are yet to be overcome to reach clinical applications. The major limitation of the NSC-based therapy is its inability to retain most of the donor stem cells after grafting into an ischemic brain area which is lacking of essential oxygen and nutrients for the survival of transplanted cells. Low cell survival rate limits the capacity of NSCs to repair the injured area and this poses a much more difficult challenge to the NSC-based therapy for ischemic stroke. In order to enhance the survival of transplanted cells, several stem cell culture preconditioning strategies have been employed. For ischemic diseases, hypoxic preconditioning is the most commonly applied strategy since the last few decades. Now, the preconditioning strategies have been developed and expanded enormously throughout years of efforts. This review systematically presented studies searched from PubMed, ScienceDirect, Web of Science, Scopus and the Google Scholar database up to 31 March 2020 based on search words containing the following terms: “precondition” or “pretreatment” and “neural stem cell” and “ischemic stroke”. The searched data comprehensively reported seven major NSC preconditioning strategies including hypoxic condition, small drug molecules such as minocycline, doxycycline, interleukin-6, adjudin, sodium butyrate and nicorandil, as well as electrical stimulation using conductive polymer for ischemic stroke treatment. We discussed therapeutic benefits gained from these preconditioned NSC for in vitro and in vivo stroke studies and the detailed insights of the mechanisms underlying these preconditioning approaches. Nonetheless, we noticed that there was a scarcity of evidence on the efficacy of these preconditioned NSCs in human clinical studies, therefore, it is still too early to draw a definitive conclusion on the efficacy and safety of this active compound for patient usage. Thus, we suggest for more in-depth clinical investigations of this cell-based therapy to develop into more conscientious and judicious evidence-based therapy for clinical application in the future.

## 1. Introduction

Stroke is defined as rapid neurological dysfunction due to the disturbance in cerebral blood supply, with acute onset of clinical signs and symptoms persisting for more than 24 h or until death. It is a leading cause of neuronal impairment and adult disability worldwide in which about 70% of the stroke survivors suffer from reduced work capacity, while the remaining 30% require self-management assistance. About 85% of the stroke cases reported are classified as ischemic stroke, a focal neurological deficit that occurs due to insufficient cerebral blood flow (CBF) triggered by occlusion of blood vessel [1]. CBF less than 20 mL/100 g of brain tissue per minute will lead to irreversible neuronal injury within an hour, whilst a complete absence of CBF within 4–10 min will cause immediate death of brain tissue [2]. The onset of ischemic neuronal injury is initiated by the disruption of brain nutrient, energy and oxygen, which elicits a series of interrelated and coordinated biochemical events that eventually lead to the neurovascular unit damage within the core of infarction. These biochemical events include ionic imperturbation, release of excess glutamate which leads to excitotoxicity, rapid increase in intracellular calcium that in turn activates multiple intracellular death pathways, accumulation of pro-inflammatory cytokines which promotes inflammatory injury and ultimately production of free radicals which leads to irreversible damage of the respiratory chain, mitochondrial dysfunction and eventually neuronal cell death [3].

The neuronal cell death that occurs following ischemic stroke can be reduced if the blood flow occlusion is removed and tissue reperfusion is restored quickly. Treatment for patients with acute ischemic stroke is guided by the time from the onset of stroke, the severity of neurologic deficit and findings on neuroimaging. It was proved that intravenous thrombolysis with intravenous recombinant tissue plasminogen activator (rtPA, alteplase) administration significantly reduced mortality and disability associated with ischemic stroke within 4.5 h after onset [4,5]. Later treatment may improve outcomes in selected patients, with the treatment window extended to 9 h from onset. Moreover, there is growing evidence that intra-arterial catheter-based mechanical thrombectomy of occluded large intracranial arteries improves outcomes in selected patients with acute ischemic stroke when performed up to 24 h after onset [6]. Nonetheless, despite the fact that these primary therapies could allow rapid restoration of blood supply and reduce stroke-related disability and mortality, they are unable to trigger regeneration of new viable neuronal cells to replace the damaged brain tissue and thus cannot completely restore the brain function [7]. As a consequence, poor prognosis is observed among ischemic stroke patients. This is indicated by the reports which showed that almost one-third of stroke patients who received the clinical stroke treatment are unable to ambulate even after treatment [8], and almost half of stroke patients who have suffered a first stroke are at risk of recurrent stroke within a few days or weeks after the initial treatment [9]. The constraints of current ischemic stroke therapy have led to the demand for alternative therapeutic approaches such as using robust neural stem cell (NSC)-based therapy to promote brain repair and remodeling in stroke-induced injury patients [10].

## 2. Neural Stem Cell (NSC) Treatment for Ischemic Stroke

**Figure 1:**
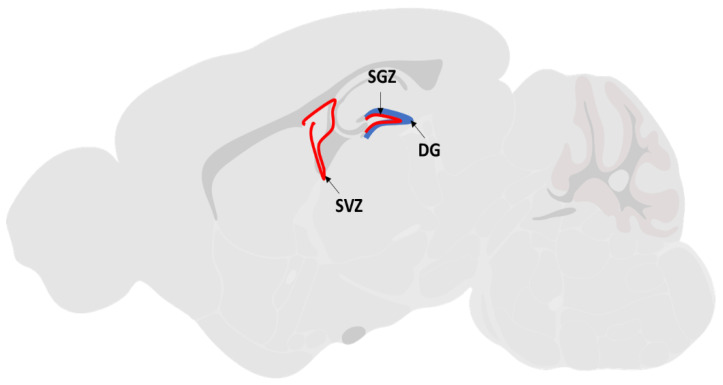
The sagittal brain section illustrated the location of the subventricular zone (SVZ) of the lateral ventricles and the subgranular zone (SGZ) of the dentate gyrus (DG) regions.

Survival, proliferation, migration and differentiation of NSC are the key regenerative processes to promote post ischemic recovery. Reports had shown that patients with ischemic stroke injury are capable of undergoing natural endogenous neurogenesis at the ischemic penumbra region of the brain [11,12,13]. Endogenous NSCs reside in two active neurogenesis brain regions: the subventricular zone (SVZ) of the lateral ventricles and the subgranular zone (SGZ) of the dentate gyrus (DG) of the hippocampus [14] (Figure 1). Under normal physiological conditions, these endogenous NSCs remain quiescent in adult brains. After ischemic injury, the endogenous NSCs are activated to proliferate and migrate towards the lesion site by several neurotrophic factors secreted by the ischemic brain tissue. The factors include brain-derived neurotrophic factor (BDNF), vascular endothelial growth factor (VEGF), cytokines like monocyte chemoattractant protein (MCP-1) and macrophage inflammatory protein (MIP-1) [14]. Within a week after the lesion, newly generated neurons appear at the boundary of the damaged area [15], implying that neuroregenerative therapy using endogenous NSCs is highly anticipated as an effective strategy for treating degenerative brain diseases such as ischemic stroke. Nonetheless, this spontaneous regeneration by endogenous NSCs is insufficient for structural or functional restoration of the injured brain, as the majority of these newly formed neurons die before functional maturation, leaving only a limited number of cells which could stably integrate into the neuronal circuitry [16].

Therefore, current NSC-based therapy not only depends on endogenous repair, but also depends on exogenous NSC transplantation from independent sources. The exogenous NSC transplantation approach exhibits therapeutic potential since it is not only capable of directly replacing the damaged brain tissue, but also able to secrete neuroprotective factors to protect brain tissue at risk during the acute phase of the stroke. Reports of exogenous NSC transplantation in ischemic stroke-induced animal models showed promising data, with conclusive efficacy and potential of this therapy. Majority of the studies reported improved prognosis of this disease after exogenous NSC transplantation such as improved neurological functions and strengthened motor skills [17]. Basically, the exogenous NSC transplantation focuses on two strategies: replace dead cells in the infarcted area or enhance the self-repair system by providing trophic support to mediate the neural network reconstruction [18]. Furthermore, the exogenous NSCs could be preconditioned prior to transplantation to enhance its therapeutic potential for ischemic stroke. Preconditioning strategies have been tested in many types of stem cells, including NSCs, to increase grafted cell survival rate and their therapeutic potential within hostile ischemic brain microenvironments. The concept of preconditioning was first described in the past 30 years. It applied the hormesis biological concept, whereby one or more brief episodes of sub-lethal stimuli was introduced to render the endogenous protective mechanisms against subsequent lethal injury [19]. In recent years, various preconditioning strategies have been developed and explored to improve donor cell survival after transplantation. Nonetheless, there is still no systematic review to report and vindicate the potential of these preconditioning strategies for exogenous NSC transplantation-based therapy. Therefore, in this study, we used a systematic review method to comprehensively search for the literature describing the efficacy of preconditioning strategies to enhance NSC therapeutic potential for ischemic stroke treatment as described in the Section 3: Methodology.

## 3. Methodology

### 3.1. Search Strategy

In this review, a literature search based on Preferred Reporting Items for Systematic Reviews and Meta-Analyses (PRISMA) guideline was performed to identify publications studying the potential of preconditioned NSC for ischemic stroke treatment conducted in vitro, in vivo or in both. Four specialized databases: PubMed, ScienceDirect, Web of Science and Scopus were used for the literature search using the keywords “precondition” or “pretreatment” and “neural stem cell” and “ischemic stroke”. The systematic search was conducted up to 31 March 2020. In addition, Google Scholar was searched too to find additional studies of interest. In order to achieve a highly sensitive search, the first 100 hits of Google Scholar sorted by relevance were included in the analysis.

### 3.2. Research Article Selection and Evaluation

Search results were limited to fully documented articles in English following the inclusion and exclusion criteria listed below:

Inclusion criteria:

i. Full-text articles
ii. In vitro studies related to preconditioned NSC for ischemic stroke therapy
iii. In vivo studies related to preconditioned NSC for ischemic stroke therapy
iv. Intervention clinical trial related to preconditioned NSC for ischemic stroke therapy

Exclusion criteria:

i. Irrelevant titles and abstracts
ii. Duplicated studies
iii. Review articles/meta-analyses
iv. News/editorials/letters
v. Case reports
vi. Non-English language

Two independent reviewers screened the articles based on the inclusion and exclusion criteria stated above. For the first screening, the related articles were screened based on their titles and abstracts. Next, the remaining papers were checked for duplications. Finally, the selected full-text articles were checked by another reviewer according to the inclusion criteria for final validation.

## 4. Result

**Figure 2:**
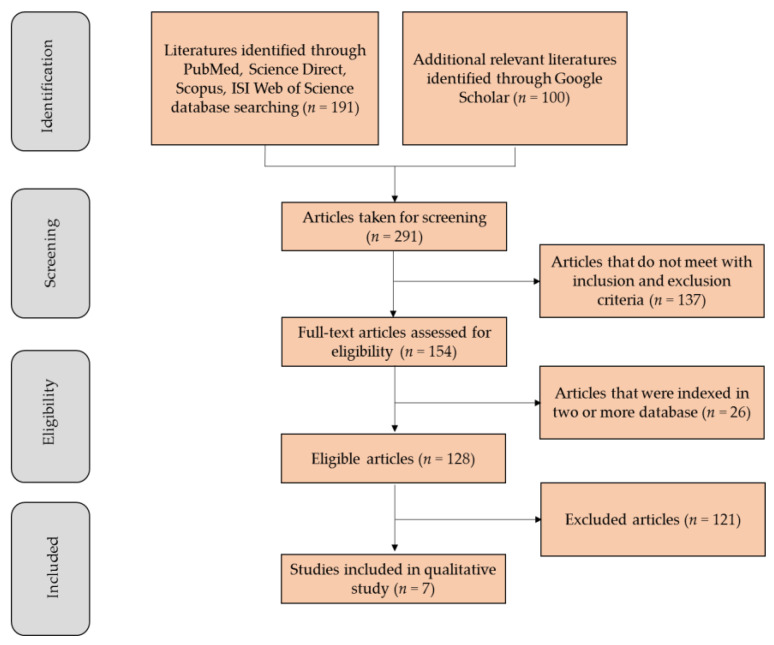
Flowchart illustrating the progressive study selection based on Preferred Reporting Items for Systematic Reviews and Meta-Analyses (PRISMA) flow Diagram Search Strategy.

Study selection: The primary search identified 191 articles, including 25 from PubMed, 138 from ScienceDirect, 24 from Web of Science and 4 from Scopus. The first 100 hits from the ‘Most Relevant’ option in Google Scholar were also added. Among these, 137 documents were published in languages other than English or not a research article that met the inclusion criteria. In addition, 26 manuscripts were indexed in two or more databases and were considered only once, resulting in 128 eligible articles. After an initial screening of titles and abstracts, followed by a full-text analysis and validation, 7 articles that fulfilled all the selection criteria were included in this systematic review. A flowchart illustrating the progressive study selection and numbers at each stage is shown in Figure 2.

## 5. Discussion

**Figure 3:**
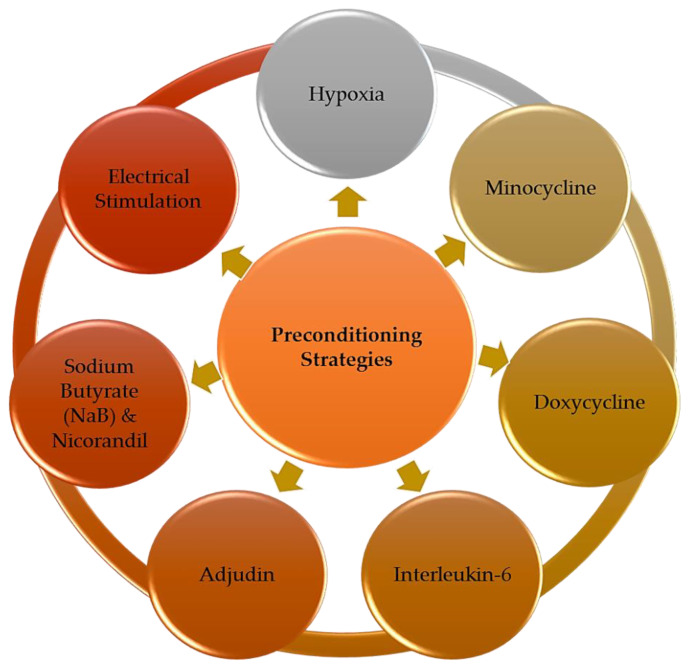
The major preconditioning strategies applied to enhance neural stem cell (NSC)-based therapy for ischemic stroke treatment, which include hypoxic condition, small molecules such as minocycline, doxycycline, IL-6, adjudin, NaB and nicorandil and electrical stimulation using conductive polymer.

The 7 articles selected were carefully read and analyzed. Based on the collected literature data, we identified seven major preconditioning strategies applied to enhance NSC-based therapy for ischemic stroke treatment (Figure 3). The therapeutic benefits gained from these preconditioned NSC for in vitro and in vivo stroke studies and the detailed insights of the mechanisms underlying these preconditioning approaches were discussed in detail in this section. The summary of all key findings reported is listed in Table 1.

### 5.1. Hypoxic Preconditioning

Oxygen (O_2_) is one of the critical factors involved in cell proliferation and morphogenesis, where its level is highly regulated in the human body. It is known that mammalian blastocyst is physiologically exposed to oxygen levels ranging from 1.5–5.3% in the reproductive tract during the early stages of embryonic development [20]. This range of oxygen concentration was found to actually increase NSC proliferation and modulate its differentiation into functional neuron, instead of the ambient oxygen level (~21%) [21]. Therefore, a low oxygen concentration environment, known as a hypoxic condition, has become a topic of interest among researchers due to its possibility to profoundly augment stem cell microenvironment to favor the success of stem cell engraftment. Hypoxic preconditioning is applied on NSCs to trigger various protective signaling pathways and increase the stem cell resilience to ischemic-derived injuries. This strategy was reported by Wang et al. in 2015 using NSCs derived from both hemispheres hippocampus of fetal rats [22]. The study revealed that the NSCs cultured under decreased oxygen environment (5%) and glucose-free medium for 2 h were able to proliferate, differentiate and survive better than NSCs cultured under ambient oxygen environment (21%). They also reported that preconditioned NSCs formed neurospheres with multi-directional neurite outgrowth from the center, indicating the expansion and migration capacity of the cells. The multidirectional expansion/migration of preconditioned NSCs is crucial for the subsequent development of neural networks to replace the damaged brain area after stroke attack.

Furthermore, the study also reported that the preconditioned NSCs showed spontaneous calcium oscillations in the neural network. Calcium oscillations are ubiquitous signal transductions present in all cells that provide efficient means to transmit intracellular biological information [23]. In neurons, the signal transduction is particularly important in the neural excitability, as well as the synaptic plasticity. Elevated neuronal excitability increases the activity of neuronal networks, resulting in the restoration of excitatory potentials of the impaired neural circuit function after ischemic stroke attack.

**Figure 4:**
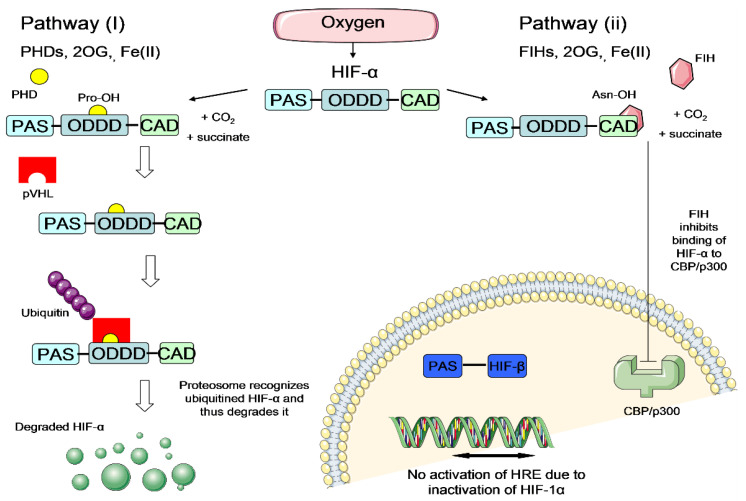
Regulation of HIF-α via two pathways under normal oxygen levels (atmospheric oxygen level~21%). Left panel: Pathway (I)—PHD enzymes hydroxylate the prolyl (Pro) residue of HIF-α in ODDD domain, in the presence of oxygen, 2OG and Fe(II). Hydroxylated HIF-α is recognized by pVHL protein which labels the HIF-α to proteasomal degradation by ubiquitin protein. Degraded HIF-α is no longer able to activate the HIF system and thus the HIF system is downregulated under normal oxygen level. Right panel: Pathway (II)—FIH enzymes hydroxylate asparagine (Asn) residue of HIF-α in the CAD domain, in the presence of oxygen, 2OG and Fe(II). Hydroxylated HIF-α is blocked to interact with the essential transcriptional coactivator CBP/p300, thus silencing the HIF transcriptional ability and downregulate the HIF system. HIF = hypoxia-inducible factor; HIF-α = hypoxia-inducible factor α-subunit; HIF-β = hypoxia-inducible factor β-subunit; PAS = Per ARNT SIM domain; ODDD = oxygen-dependent degradation domain; CAD = C-terminal transactivation domain; PHD = prolyl hydroxylase; FIH = factor inhibiting HIF; 2OG = 2-oxoglutarate; Fe(II) = iron ferrous; Pro = prolyl; Asn = asparagine; pVHL = von Hippel Lindau protein; CBP/p300 Creb-binding protein/protein 300; HRE = hypoxia response elements.(Figure source: Tan, S.C., Cardiac Stem Cell Therapy for Infarcted Rat Hearts, in the Department of Physiology, Anatomy and Genetics. 2011, University of Oxford: Oxford. p. 226.) [32].

**Figure 5:**
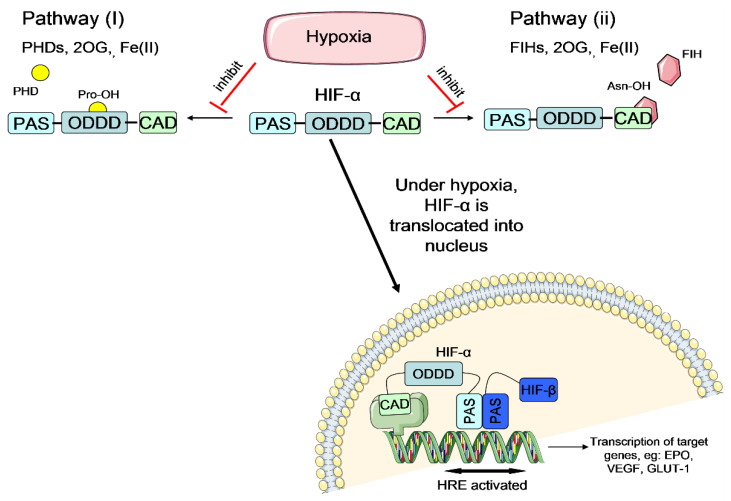
Regulation of HIF-α via two pathways under hypoxia (deprived oxygen condition~0–5% O2). Under hypoxia both pathway (I) and pathway (II) are inhibited due to absence of oxygen (the essential substrate of PHD and FIH enzyme). Therefore, HIF-α subunit escapes the hydroxylation by PHD or FIH enzymes, and subsequently translocates to the nucleus and dimerises with HIF-1β subunit to form functional heterodimeric HIF. Activated HIF i then binds to the hypoxia response elements (HREs), leading to a transcriptionally active complex which activates the expression of hundreds of genes including EPO, VEGF and GLUT-1. HIF = hypoxia-inducible factor; HIF-α = hypoxia-inducible factor α-subunit; HIF-β = hypoxia-inducible factor β-subunit; PAS = Per ARNT SIM domain; ODDD = oxygen-dependent degradation domain; CAD = C-terminal transactivation domain; PHD = prolyl hydroxylase; FIH = factor inhibiting HIF; 2OG = 2-oxoglutarate; Fe(II) = iron ferrous; Pro = prolyl; Asn = asparagine; HRE = hypoxia response elements; EPO = erythropoietin; VEGF = vascular endothelial growth factor; GLUT-1 = glucose transporter 1.(Figure source: Tan, S.C., Cardiac Stem Cell Therapy for Infarcted Rat Hearts, in the Department of Physiology, Anatomy and Genetics. 2011, University of Oxford: Oxford. p. 226.) [32].

It is believed that the hypoxic preconditioning enhances NSC survival rate under ischemic condition mainly through the activation of hypoxia-inducible factor (HIF) transcriptional complex [24,25]. HIF transcriptional complex comprises of HIF-α and HIF-β subunits. It is the master regulator that mediates essential homeostatic responses to reduced oxygen availability in mammals. In the presence of oxygen, the HIF-α subunit is downregulated via two pathways: hydroxylation by prolyl-4-hydroxylase (PHD) enzymes or hydroxylation by factor-inhibiting HIF (FIH) enzymes [26,27] (Figure 4). These hydroxylations inhibit the activation of the HIF system in the presence of oxygen. On the other hand, under hypoxia, the HIF-α subunit escapes the hydroxylation by PHD or FIH enzymes, translocates to the nucleus and dimerises with the HIF-1β subunit to form functional heterodimeric HIF (Figure 5). Activated HIF is then bound to the hypoxia response elements (HREs), leading to a transcriptionally active complex which activates the expression of many important genes which could enhance NSC survival under ischemic condition (Figure 5). Examples of the important HIF-regulated genes include erythropoietin (EPO), which regulates production of red blood cells [28], vascular endothelial growth factor (VEGF), which triggers neovascularisation [29] and glucose transporter 1 (GLUT-1), which facilitates uptake of glucose by cells [30]. The increase of EPO and VEGF signalling could possibly trigger new blood vessel regeneration and help to restore blood flow to the injured brain area, whilst the increase of GLUT-1 could enhance the cell affinity to nutrients. With improved survival rate of preconditioned cells, they could repopulate at the ischemic brain area and release adenosine triphosphate (ATP), D-serine and glutamate (known as gliotransmitters), allowing the transplanted cells to improve the plasticity, excitability and synaptic activity for brain function restoration [31]. Thus, it is hypothesized that hypoxic preconditioning could increase the therapeutic potential of NSCs to treat stroke disease.

### 5.2. Minocycline

Minocycline, a semisynthetic second-generation tetracycline derivative, is an antibiotic used to combat bacterial infection. Besides being used as an antibiotic, minocycline also exhibits anti-oxidant, anti-inflammatory and anti-apoptosis effects in various experimental models of acute neurological injury [33]. Known for its highly lipophilic feature, superior blood-brain barrier penetration ability and neuroprotective effects at an extended time window (more than 3 h), minocycline has been tested in animal models of hemorrhagic and ischemic stroke, Parkinson’s disease, spinal cord injury, Huntington’s disease and many other neurological related diseases [34,35].

Preconditioning of NSCs using minocycline for ischemic stroke therapy has been demonstrated by Sakata and colleagues in 2012 [36]. They isolated NSCs from the subventricular zone (SVZ) of fetal rats, preconditioned with 10 µM minocycline for 24 h and transplanted into cerebral ischemia rat models induced via transient middle cerebral artery occlusion (MCAO) surgery. The study revealed that preconditioning with minocycline successfully protected the grafted NSCs from ischemic reperfusion injury via upregulation of nuclear factor erythroid 2 (Nrf2) and Nrf2-regulated antioxidant genes included NADPH quinone dehydrogenase 1 (NQO1) (where NADPH = nicotinamide adenine dinucleotide phosphate) and heme oxygenase-1 (HO-1). The Nrf2 is a major regulator of antioxidant and cellular protective genes, which is primarily activated in response to oxidative stress. Under normal physiological conditions, Nrf2 is constitutively expressed in the cytoplasm of cells at a low level. However, when a cell is exposed to oxidative stress, for example in an ischemic stroke brain, the Nrf2 will be translocated into the cell nucleus and bound to the antioxidant response element (ARE) genes that encode for antioxidant proteins to protect the cells from elevated reactive oxidative species (ROS) and free radicals [37]. The protective nature of Nrf2 conferred significant cytoprotection effects on the minocycline-preconditioned NSCs in which they showed reduced grafted-cell death by 76% and extensive migration towards the ischemic lesion border, after transplanted into the MCAO ischemic stroke rat models. Moreover, these grafted NSCs also were found to successfully proliferate and differentiate into neurons, astrocytes or oligodendrocytes 28 days after transplantation as reported by Sakata et al. [36].

In addition, the transplantation of minocycline-preconditioned NSCs also was found to facilitate neuroprotection effects in ischemic stroke rats via the upregulation of neurotrophic factors such as (1) glial cell-derived neurotrophic factor (GDNF), which potentially promotes the survival of many types of neurons; (2) brain-derived neurotrophic factor (BDNF), which supports survival of existing neurons and encourages growth and differentiation of new neurons and synapses; (3) nerve growth factor (NGF), which primarily involves in the regulation of growth, maintenance, proliferation and survival of neurons; and (4) VEGF cytokine, which triggers neovascularization. The neuroprotective effects exerted by these neurotrophic factors and cytokines were indicated by the attenuated infarct size and improved functional/behavioral recovery in animals transplanted with the minocycline-preconditioned NSCs, compared to the non-transplanted animal group. Moreover, Sakata and colleagues also proved that these factors were released by minocycline-preconditioned NSCs due to the upregulation of Nrf2. This is because the minocycline-induced neuroprotection was abolished by transfecting the NSCs with Nrf2-small interfering RNA which can block the expression of Nrf2.

**Figure 6:**
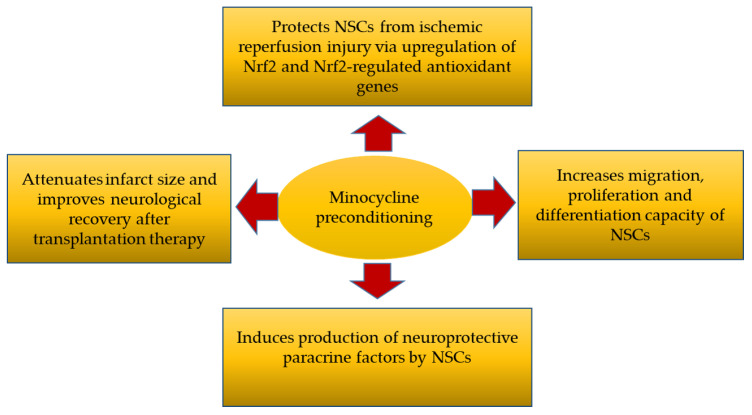
Four major mechanism of action by minocycline-preconditioned NSCs after transplanted into ischemic stroke rat models [36].

In brief, this study revealed that preconditioning with minocycline (1) protected NSCs from ischemic reperfusion injury via upregulation of Nrf2 and Nrf2-regulated antioxidant genes, (2) increased the migration, proliferation and differentiation capacity of the NSCs, (3) induced the production of neuroprotective paracrine factors by NSCs and (4) resulted in attenuated infarct size and improved neurological recovery after NSC transplantation into ischemic stroke rat models (Figure 6).

### 5.3. Doxycycline

Similar to minocycline, doxycycline is a tetracycline-derived antibiotic mainly used against microbial infections. It has prolonged serum half-life between 12 and 25 h that allows fewer daily doses usage, making it safer for patients with renal failure [38]. It also has better characterized safety profiles and lesser known adverse effects compared to other members in the tetracycline family [39]. Doxycycline is useful for the treatment of infectious-related diseases such as respiratory tract, genitourinary and spirochetal infection. It is also frequently administered for non-infectious conditions such as severe acne, periodontitis, rheumatoid arthritis, neutrophilic diseases and multiple sclerosis [40].

Since doxycycline is a member of second-generation tetracycline, it shares similar chemical structures and mechanism of actions as minocycline. This antibiotic was tested as a potential NSC preconditioning agent in 2013, where Yousra and colleagues confirmed the effectiveness of this small molecule as a potential strategy to enhance the NSC therapeutic potential for ischemic stroke treatment [41]. This study isolated NSCs from bilateral SVZ of fetal rat brains and preconditioned the NSCs with doxycycline at 8 uM for 24 h. They found that the doxycycline preconditioning significantly reduced the cell apoptosis in an in vitro cerebral ischemic model measured using lactate dehydrogenase (LDH) and terminal deoxynucleotidyl transferase nick end labeling (TUNEL) assays. LDH is an oxidoreductase enzyme found in nearly all living cells to catalyze the interconversion of lactate to pyruvate during cell metabolism. Under normal physiology, LDH remains within cytoplasm, however, when a cell is undergoing injury or cell death, the LDH is released into the bloodstream, where it is identified higher than normal levels. Therefore, LDH level is very often measured to evaluate the presence of cell damage or death. On the other hand, TUNEL assay is a method to detect DNA fragmentation by labeling the 3′- hydroxyl termini in the double-strand DNA breaks generated during apoptosis. The higher the signal indicates the higher the apoptosis rate. In addition, Yousra et al. also reported that the cytoprotective effect induced by doxycycline preconditioning was related to the activation of Nrf2 transcription factor that regulates the expression of antioxidant genes. The finding was supported by reduced superoxide anion (O_2_^•−^) production in doxycycline-preconditioned NSCs, compared to non-preconditioned NSCs. O_2_^•−^ is a radical species with exceptionally high reactivity due to its unpaired electron. The presence of O_2_^•−^ could cause cytotoxicity and damage to DNA [42]. Therefore, the ability of doxycycline to enhance Nrf2 expression to scavenge the O_2_^•−^ resulted in higher survival of NSC in ischemic stroke models.

In summary, this study provided in vitro evidence that doxycycline preconditioning reprograms NSCs to tolerate oxidative stress and results in reduced apoptosis and increased cell viability, making the cells a better therapeutic agent for ischemic stroke treatment.

### 5.4. Interleukin-6

Interleukin-6 (IL-6) is a pleiotropic cytokine that plays a crucial role in the pathogenesis of various autoimmune and neurological disorders, including the ischemic stroke. IL-6 is produced by both lymphoid and non-lymphoid cells, causing an antigen-specific immune response and excessive inflammation process that may aggravate ischemic cerebral damage [43]. However, aside from its pro-inflammatory harmful effects, IL-6 also has been reported to promote activation of intrinsic survival signalling, offering its neuroprotective effects for ischemic brain [44]. This mechanism, known as signal orchestration model, displays how IL-6 can be either a pro-inflammatory or an anti-inflammatory molecule [45].

Based on this knowledge, Sakata and colleagues (2012) performed a series of experiments to elucidate the exact role of IL-6 in NSC preconditioning strategy [46]. To do that, they isolated NSCs from bilateral SVZ of fetal mouse brains and preconditioned the NSCs with 20 ng/mL IL-6 for 24 h. They found that this preconditioning strategy successfully reprogrammed NSCs to tolerate ischemic injury through the activation of signal transducer and activator of transcription 3 (STAT3). STAT3 activation induced the expression of superoxide dismutase 2 (SOD2), a mitochondrial antioxidant enzyme which plays an anti-apoptotic role against oxidative stress and inflammatory cytokines [47]. This evidence was supported by in vitro Western blot and immunochemistry data, where the NSCs preconditioned with IL-6 for 24 h significantly upregulated SOD2 enzyme expression, along with an increase of STAT3 expression level that was predominantly distributed in the nucleus. Moreover, when the NSCs were subjected to the oxidative stress stimuli such as hydrogen peroxide (H_2_O_2_) and diethylenetriamine/nitric oxide, the IL-6 preconditioned NSCs survived better (by 28%) compared to the non-preconditioned NSCs, indicating the cytoprotection effects exerted by IL-6 preconditioning. Taken together, these findings confirmed the pivotal role of IL-6 as a potential anti-oxidant agent to protect NSCs against the ischemic reperfusion injury.

Furthermore, IL-6 preconditioning has been reported to significantly increase the tolerance of treated cells against apoptotic processes and hostile environments in vivo. NSCs subjected to IL-6 pre-treatment demonstrated a reduction in grafted-cell death by 62% when transplanted into ischemic stroke mouse models. Moreover, these grafted cells managed to survive in the ischemic brains up to 28 days, with no formation of tumors. Staining using green fluorescent protein (GFP) also revealed extensive grafted cell migration toward the ischemic lesion borders, where the grafted cells were capable of differentiating into neurons, oligodendrocytes and astrocytes. More excitedly, the cortical infarct volume was significantly attenuated in the preconditioned NSC group compared with the non-preconditioned control group. These findings indicated that IL-6 preconditioning successfully increased the survival rate of grafted cells and induced their integration into the impaired neuronal networks after ischemic stroke injury.

According to Sakata et al. [46], the enhancement of grafted cell survival and integration into neuronal networks could be due to the enhanced angiogenesis induced by expression of VEGF in IL-6 preconditioned NSCs. This was supported by the rapid increase of blood vessel density in animals transplanted with IL-6 preconditioned NSCs. Restoration of vascular and blood supply after ischemic injury is very crucial for post-stroke neuroregeneration. Numerous studies have shown that angiogenesis is positively correlated to the survival rate of stroke patients, indicating that modulation of the vascular growth in the ischemic area could be an important therapeutic target for ischemic stroke [48]. Indeed, in this study, angiogenesis had been found to be closely related to significant functional recovery in the IL-6 preconditioned NSC group, as compared to the non-preconditioned NSC group. In conclusion, this study indicated that IL-6 preconditioning successfully enhanced the therapeutic potential of NSCs for ischemic stroke by increasing anti-oxidant activities, improving the survival rate of grafted cells, inducing their integration into the impaired neuronal networks, and elevating angiogenesis after ischemic stroke injury.

### 5.5. Adjudin

Adjudin is an indazole-based compound that has been explored as a non-hormonal male contraceptive since the 1990s. Recently, the studies on adjudin have been expanded to evaluate its biological potentials for other ailments such as anti-cancer, anti-inflammation, anti-neurodegeneration and anti-ototoxicity activities [49]. This small molecule also has been tested as one of the preconditioning strategies of NSCs to increase the cell survivability and expand the therapeutic effect of NSC-based stroke therapy by Zhang et al. (2017) [50].

According to the study, preconditioning of NSCs with adjudin at 10 and 30 uM for 24 h showed significant reduction in apoptosis rate when exposed to oxidative stress stimuli, compared with non-preconditioned NSCs. The underlying mechanism of adjudin-induced cytoprotection against oxidative damage was supported by the activation of serine/threonine-specific protein kinase (AKT) signalling pathway in adjudin-preconditioned NSCs. AKT signalling is a vital signal transduction pathway that promotes cell survival and growth in response to extracellular signals [51]. When activated, the AKT signalling acts to enhance the transcription of anti-apoptotic genes whilst inhibit the transcription of cell death genes, conferring cytoprotection to the NSCs.

Adjudin also exhibited potential to enhance the expression of antioxidant genes, which plays important roles in resistance to oxidative stress. The antioxidant genes expressed in adjudin-preconditioned NSCs were SOD2, catalase and glutamate-cysteine ligase catalytic (GCLC). Moreover, adjudin pre-treatment was also able to inhibit the expression of inducible nitric oxide synthase (iNOS), compared to non-preconditioned NSCs. Induction of the high-output iNOS usually occurs in an oxidative stress environment, for example, following an ischemic stroke injury [52]. iNOS also plays role as a mediator of inflammation responses in various cell types. The presence of iNOS could deteriorate inflammatory response and apoptosis. Therefore, the ability of adjudin-preconditioning to inhibit iNOS expression could protect the NSCs from excessive cell death.

Adjudin’s potential as a neuro-inflammatory modulator also has been demonstrated in this study, where it could block microglial activation, as indicated by less CD11b signal detected by fluorescence microscopy. Besides, the adjudin-preconditoned NSCs also could aid to reduce the inflammatory mediators such as interleukin-1β (IL-1β), interleukin-6 (IL-6) and tumor necrosis factor-α (TNF-α) which were dramatically increased after the ischemic injury in mouse models. It is known that an imbalance or overexpression of pro-inflammatory cytokines can lead to different pathological disorders [53]. Therefore, transplantation of adjudin-preconditioned NSCs to reduce the expression of pro-inflammatory cytokines can potentially prevent the cascade events of severe neuro-inflammatory responses, and thus be able to preserve the brain from extensive tissue damage.

Besides oxidative stress and neuroinflammation, Zhang et al. [50] also focused on the protective effects of adjudin-preconditioned NSCs on blood-brain-barrier (BBB) permeability. This is because maintaining BBB integrity is critical for reducing secondary brain injury following cerebral ischemia. They found that adjudin preconditioning could reduce the leakage of BBB by maintaining the protein levels of tight junction protein-1 (ZO-1) and occludin, which are located in the tightly sealed monolayer of brain endothelial cells (BEC) and conferred barrier function to preclude non-related blood substances permeating into the brain parenchyma [54]. This protective effect was due to the attenuation of neuroinflammatory response and oxidative stress after transplantation of adjudin-preconditioned NSCs.

The study also showed that adjudin preconditioning induced higher expression of neurotrophic factors such as BDNF, NGF and GDNF, as compared to non-preconditioned NSC. These neurotrophic factors could act to support the survival and growth of neurons. Besides, pre-treated NSCs were also able to promote angiogenesis after transplanted into ischemic stroke mouse model, measured by increased endothelial progenitor cell marker CD31 and increased new blood vessels generation. Angiogenesis is directly linked to neurogenesis because re-establishment of blood circulation in the damaged area is necessary for new neuronal survival and development. This statement is supported by the observation that adjudin-pretreated NSCs greatly reduced infarct volume by as much as 20% compared to untreated NSCs. Meanwhile, animals that received adjudin preconditioned NSCs also showed improved behavioural performance by 25% better than the animals treated with non-preconditioned NSCs.

Taken together, this study provided ample preclinical experimental evidences to confirm that adjudin preconditioning is able to enhance the survival rate of NSCs and exhibit better effect on inhibiting oxidative stress and neuroinflammation, maintaining BBB integrity, expressing higher levels of neurotrophic and angiogenesis factors after transplanted into ischemic stroke model. All these effects resulted in stronger therapeutic effects of NSCs for ischemic stroke brain injury treatment, indicated by decreased infarct volume and improved behavioral outcomes in the ischemic stroke animal models.

### 5.6. Sodium Butyrate and Nicorandil

Sodium butyrate (NaB) is a functional molecule produced from microbial fermentation in the mammalian intestine [55]. NaB-based compounds have been found to exert neuroprotective effects and thus have been explored to treat various neurological diseases such as Huntington’s disease, spinal muscular atrophy and Parkinson’s disease [56]. On the other hand, nicorandil is an orally efficacious anti-anginal medication characterized by nitrate moiety in its chemical structure [57].Recent findings indicated that the novel combination of these two distinct drugs conferred neuroprotective effects against ischemic-associated neuronal injury [58], offering its potential as prospective treatment in ischemic stroke.

Preconditioning of NSCs using NaB combined with nicorandil for stroke therapy has been explored by Seyed and colleagues in 2018 [58]. In this study, fetal mouse ganglionic NSCs were isolated, preconditioned by combined treatment of NaB and nicorandil, and transplanted in an experimentally induced stroke mouse model. NSCs preconditioned with NaB or nicorandil alone were used as controls. They found that NSCs preconditioned using NaB or nicorandil alone were able to increase BDNF expression in vitro and post-engraftment transplantation. However, the combined treatment (NaB + nicorandil) increased BDNF expression significantly higher than the single treatment, indicating the synergistic effects of combination treatment compared to single treatment. BDNF is a neurotrophic factor that is responsible for the neurogenesis process and survival signalling for various types of neurons. Therefore, it is not surprised that the combo treatment of NaB + nicorandil resulted in the highest survival rate of transplanted cells (13.6 ± 4.59 cells/mm^2^), as compared to cells preconditioned with either NaB (7.4 ± 4.9 cells/mm^2^) or nicorandil (8 ± 3.7 cells/mm^2^) alone.

Besides that, they also observed that induction of BDNF in the preconditioned NSCs led to an increase in phosphoinositide 3-kinases (PI3K) activity. PI3K is an enzyme involved in cellular functions such as cell growth, proliferation, differentiation, motility, survival and intracellular trafficking. As reported in previous studies, PI3K/Akt signaling stimulates neurite outgrowth [59]. In accordance to this finding, transplantation of NSCs preconditioned using NaB and nicorandil also significantly increased the length of neurite outgrowth and showed a better outcome in infarct size reduction (50%) and neurological performance at the end of transplantation study.

Anti-inflammatory properties of NaB and nicorandil-preconditioned NSCs on ischemic stroke also were demonstrated in this study, where the preconditioning strategy was found to sucessfully inhibit pro-inflammatory mediators (IL-1β, IL-6, IL-12, and TNF-α) and upregulate anti-inflammatory mediators (IL-10). Besides, this preconditioning strategy is also able to suppress microglial activation. Suppression of microglial activation is critical to inhibit severe neuroinflammatory responses which could contribute to the progression of many chronic neurodegenerative disorders [60].

In summary, this study reported that NSC preconditioning by combined treatment using small molecules NaB and nicorandil enhances the post-engraftment survival rate, increases the expression of neurotrophic factor, increases neurite outgrowth, reduces inflammatory responses, and is thus able to potentiate their reparability in a rodent model of experimental stroke, indicated by the reduced infarct size and improved behavioral performance.

### 5.7. Electrical Stimulation Using Conductive Polymer

Recently, electrical stimulation has garnered interest as an effective physical stimulus to manipulate cell behaviors in vitro and in vivo [61]. There are increasing evidences to prove that electric stimulation can activate many intracellular signaling pathways and influence the intracellular microenvironment [62,63]. For example, Prabhakaran et al. (2011) [64] proved that electrical stimulation of nerve stem cells on specific conductive polymer can stimulate the differentiation of nerve stem cells and neurite elongation, highlighting the relevant application of electrical impulses as target preconditioning strategy for nerve tissue regeneration.

Conductive polymer is a form of biomaterial that has gained more and more attention in cell biology and neural tissue engineering applications due to its electrical excitability. This biomaterial has great biocompatibility and is able to interact continuously with the applied stem cells and their surrounding neural environment, making the NSC a better candidate for ischemic stroke therapy [65]. Previously, electrically conductive biomaterial was commonly made of carbon nanotubes, graphene and inorganic particles such as gold and silver nanoparticles [66,67]. However, the safety of these conventional materials is in doubt because these materials are not biodegradable and thus can cause long term in vivo toxicity such as chronic inflammation and compression of nerves over time. Therefore, alternative conductive biomaterials made up by polypyrrole (PPy), polyaniline (PANi), and poly (3,4-ethylenedioxythiophene) (PEDT, PEDOT) [68,69] are introduced as an alternative to alleviate this problem. In 2017, George and colleagues developed an electrical conductive polymer scaffold made of PPy to precondition NSCs derived from H9 human embryonic stem cell line [70]. To do this, they seeded the NSCs on the PPy scaffold attached to electric wires supplied with +1 V to −1 V square wave at 1 kHz for 1 h. Then, they removed the preconditioned cell chamber from the scaffold and implanted it onto the cortex of rats that underwent distal middle cerebral artery (dMCA) occlusion, which mimics the ischemic stroke condition.

In the study, they reported electrical conductive polymer priming the preconditioned NSCs to promote functional recovery via modulation of paracrine factor vascular endothelial growth factor A (VEGF-A). The activation of the VEGF-A pathway increased blood vessel density via the angiogenesis process, validating its function to restore blood supply to the affected brain. Animal models that received NSCs preconditioned using this strategy also experienced faster and advanced functional recovery as compared to the animal group that received unstimulated NSCs. Interestingly, electrically preconditioned NSCs were not only able to improve the rate of recovery, but also able to enhance long-term function as they found that rats that received electrically preconditioned cells continued to perform better throughout their testing, indicating a long-lasting effect of in vitro electrical preconditioning.

## 6. Limited Application of Preconditioned-NSC in Clinical Ischemic Stroke Therapy

Data retrieved from Cochrane library, U.S. Clinical Trial Database (clinicaltrials.gov) and Chinese Clinical Trial Registry recorded only a few numbers of trials using non-preconditioned NSC as clinical cell lines in ischemic stroke treatment (summarized in Supplementary Table S1. The first exogenous NSC transplantation on clinical patients with brain ischemic stroke was performed in 2010, where the study published their findings in the Lancet issue (August 2016) [71]. The results from this two years study revealed safety and promising outcome of exogenous transplantation of NSCs in stroke, with no adverse effects and improved neurological functions, as indicated by the National Institutes of Health Stroke Scale (NIHSS) and the Barthel Index (BI). However, since the study involving a small number of patients, with no placebo or control groups, Phase II clinical trials were conducted with more detailed data in 2014. Most of the clinical trials are still currently ongoing and actively recruiting the patients, therefore, the efficacy and safety of this treatment is still not validated. Moreover, since the clinical application of NSC therapy is still at an early stage, none of the clinical data recorded use pre-treated or preconditioned NSCs as described in earlier sections. Due to the limited clinical data available regarding application of preconditioned-NSC for ischemic stroke therapy, it is still too early to draw a definitive conclusion on the efficacy and safety of this active compound for patient usage.

## 7. Recommendations and Future Perspective of Preconditioned-NSC Therapy

Despite the fact that limited clinical data is available to date, NSCs preconditioned by hypoxic condition, small molecules such as minocycline, doxycycline, adjudin, IL-6, combination of NaB and nicorandil, as well as electrical stimulation had been found to significantly improve cell survival and biological functions of transplanted cells in experimental scenarios. From the largely flourishing outcomes in the experimental models, preconditioned NSC definitely possesses potential to be applied in clinical treatments, provided all the issues and impracticalities are effectively addressed and solved in future.

Besides, we noticed that the preconditioning methods discussed in this article involved NSCs isolated from different resources, for example, NSCs derived from hemispheres hippocampus of fetal rat brains (hypoxic preconditioning) [22], NSCs harvested from SVZ of fetal rat brains (minocycline [36] and doxycline [41] preconditioning), NSCs harvested from SVZ of fetal mice brains (IL-6 preconditioning) [46], NSCs harvested from bilateral cortex of mouse brains (adjudin preconditioning) [50], NSCs harvested from mouse 14-day-old embryo ganglion eminence (combined NaB and nicorandil preconditioning) [58], and human neural progenitor cells (hNPCs) derived from H9 human embryonic stem cell line (electrical preconditioning) [70]. Each of these NSC types has a different origin and its own unique characteristics. Therefore, further investigations are required to determine the effectiveness and consistency of these preconditioning strategies on different NSC types, as well as the optimum timing of the treatment. This research gap could be further explored in future by researchers in related field.

Another important aspect that needs to be considered for preconditioned NSCs-based therapy is its long term safety, particularly with regard to the potential of tumorigenicity after stimulation by preconditioning approaches. In order to further enhance the efficacy of stem cell-based transplantation therapy, a combination of NSC engineering strategies with preconditioning strategies is recommended to provide enhanced cell replacement, trophic support, immunomodulatory as well as regenerative potential for better therapeutic effects. In addition, alternative treatments using NSC-derived extracellular vesicles (EV) have also been highlighted recently in neurodegenerative disease. This EV-based therapy has spurred a renewed interest in their utility for a cell-free therapeutics option, where it could avoid problems encountered by NSCs-based therapy such as low cell survival rate and high graft rejection after transplantation.

## 8. Conclusions

Recent significant progression of NSC-based transplantation therapy has brought it to the forefront of regenerative treatment for ischemic stroke. This cell-based therapy has garnered significant interest as a potential therapeutic agent to compensate the damaged neuronal cells after ischemic injury. Some clinical trials are already underway to study the effects of NSCs transplantation for ischemic stroke, however, there are still many challenges to be optimized before NSCs therapy can be applied clinically. One of the biggest limitations of NSC-based transplantation therapy is the limited survival rate of transplanted cells. The extensive cell apoptosis post-transplantation is mainly due to the deficient oxygen and nutrient availability, mechanical stress and the ongoing acute inflammatory response at the site of the injured tissue. Among the strategies proposed to enhance the NSC therapeutic potential for ischemic stroke therapy is via the preconditioning approaches. This review outlines the preconditioning strategies that had been taken to make post-engraftment stem cell survival and regenerative therapy a closer-to-clinical reality. The preconditioning strategies discussed here were the hypoxic condition, small molecules such as minocycline, doxycycline, IL-6, adjudin, NaB and nicorandil and electrical stimulation using conductive polymer. We compiled concrete in vitro and in vivo evidence endorses that these preconditioning strategies reprograms the NSCs to tolerate oxidative stress, secrete neurotrophic factor, enhance neuroprotection, induce angiogenesis, reduce neuroinflammatory responses, reduce infarct volume and increase neurological function after engraftment. We also identified several key pathways involved in mediating the preconditioned NSCs to exhibit enhanced therapeutic potential for stroke treatment. Among the important pathways discussed here were HIF transcriptional complex, STAT3, AKT and PI3K pathways. All these pathways play significant role in regulating critical cellular biology response and are worthwhile to be further explored in the future. In conclusion, all the preconditioning strategies discussed in this review article exhibited promising potential in the enhancement of NSCs-based transplantation therapy for ischemic stroke. However, there is still a scarcity of evidence on the efficacy of these preconditioned NSCs in human clinical studies, therefore, it is still too early to draw a definitive conclusion on the efficacy and safety of this active compound for patient usage. Thus, future studies on preconditioned NSC-based transplantation therapy are imperative in the ongoing quest for an effective, feasible and safe clinical approach.

## Figures and Tables

**Table 1:** Summary of cell source, preconditioning strategy and key outcomes of studies using preconditioned NSC for in vitro and in vivo ischemic stroke therapy.

Author, Year	Ref.	Cell Source	Preconditioning Strategy	Key Outcomes	
				In Vitro	In Vivo
Wang et al., 2015	[22]	NSCs derived from hemispheres hippocampus of fetal rat brains	Preconditioned with 5% oxygen (hypoxia) and glucose-free medium for 2 h	Increased cell viability Improved size and morphology of NSCs neurospehere Maintained the capability to migrate and form neural network Enhanced spontaneous calcium oscillation for signal transduction process Promoted NSCs differentiation and neurite outgrowth	N/A
Sakata et al., 2012	[36]	NSCs harvested from SVZ of fetal rats	Preconditioned with 10 µM minocycline for 24 h	Upregulated Nrf2 and Nrf2-regulated antioxidant genes such as NQO1 and HO-1 Induced higher level of paracrine factor expression such as BDNF, NGF, GDNF, VEGF	Reduced grafted-cell death Increased cell proliferation, migration and differentiation towards ischemic lesion border Reduced infarct size Improved neurological performances
Yousra et al., 2013	[41]	NSCs harvested from SVZ of fetal rats	Preconditioned with 8 µM doxycycline for 24 h	Decreased cell apoptosis Induced higher expression of Nrf2 antioxidant genes Increased cell viability Reduced oxidative stress by decreased superoxide anion production	N/A
Sakata et al., 2012	[46]	NSCs harvested from SVZ of fetal mice	Preconditioned with 20 ng/mL IL-6 for 24 h	Activated STAT3 Upregulated anti-oxidant SOD2 expression Reduced cell death by 28% Increased VEGF levels	Reduced grafted-cell death by 62% Extensive cell migration and differentiation to ischemic lesion borders Induced secretion of VEGF and promoted angiogenesis Induced integration of grafted cells into the impaired neuronal networks Improved neurological performance
Zhang et al., 2107	[50]	NSCs harvested from bilateral cortex of mouse brains	Preconditioned with 10 and 30 µM adjudin for 24 h	Reduced apoptosis rate Activated Akt signalling pathway Enhanced the expression SOD2, catalase and GCLC antioxidant genes Inhibited iNOS expression	Blocked microglial activation Reduced pro-inflammatory cytokines such as IL-1β, IL-6, TNF-α Reduced BBB leakage Enhanced neurotrophic factors expression such as BDNF, NGF and GDNF Promoted angiogenesis Decreased infarct volume by 20% Improved behavior performance
Seyed et al., 2018	[58]	NSCs harvested from 14-day-old mice embryo ganglion eminence	Preconditioned with a combination of 1 mM sodium butyrate and 12 µM nicorandil for 1 week	Induced BDNF expression Activated P13K activity	Increased BDNF level Activated P13K activity Improved donor cell survival Higher neurite outgrowth Suppressed microglial activation Reduction of proinflammatory cytokines Reduced infarct size Enhanced neurological function
George et al., 2017	[70]	Human neural progenitor cells (hNPCs) derived from H9 human embryonic stem cell line	Preconditioned with electrical stimulation for 1 h	Increased VEGF-A expression	Increased blood vessel density Improved functional recovery

## Supplementary Materials

The following are available online at https://www.mdpi.com/2076-3425/10/11/893/s1, Table S1: Compilation of clinical trials of conventional NSC-based therapy for ischemic stroke (data searched up-to-date 31/3/2020).
