# Supplementary material for: Preconditioning Strategies to Enhance Neural Stem Cell-Based Therapy for Ischemic Stroke
Source: Brain Sci. 2020 Nov 23;10(11):893. doi: 10.3390/brainsci10110893 (PMC7700351; doi:10.3390/brainsci10110893)
Supplement: Supplementary file 1 [file brainsci-10-00893-s001.pdf]

Table S1. Compilation of clinical trials of conventional NSC-based therapy for ischemic stroke (data searched up-to-date 31/3/2020).

Title & Trial number	Study phase & Cell Source	Eligibility Criteria	Primary Outcome Measure	Secondary Outcome Measure	Evaluation Time Frame & Duration of Study	Location
Pilot Investigation of Stem Cells in Stroke (PISCES) (NCT01151124)	Phase I & NSC (CTX0E03)	• Males aged 60 years or over<br>• Unilateral ischemic stroke<br>• Modified National Institutes of Health Stroke Scale (NIHSS) score minimum 6<br>• Neurologically stable for 2 m<br>• mRS of 2-4<br>• Fit for general anaesthesia, neurosurgery and have capacity to consent<br>• Infarct at least 1cm diameter	• Adverse events	• Barthel Index (BI)<br>• Mini-Mental State Examination (MMSE)<br>• Modified Rankin Score (mRS)<br>• EuroQol-5 Dimension (EQ-5D)	1 year & 2010.6 to 2023.3 (Active/ not recruiting)	United Kingdom
Phase I Clinical Study of Intracerebral Transplantation of Neural Stem Cells for the Treatment of Ischemic Stroke (NCT03296618)	Phase I & NSC (NSI-566)	• Have the ability to understand the requirements of the study, provide written informed consent and authorization for the use and disclosure of Protected Health Information (PHI)<br>• Men and women 30-65 years old<br>• Women must have a negative serum pregnancy test and practice an acceptable method of contraception or be of non-childbearing potential<br>• At least 3 months but no more than 24 months from time of stroke, with a motor neurological deficit<br>• Documented history of completed ischemic stroke in subcortical region of middle cerebral artery or lenticulostriate artery with or without cortical involvement<br>• mRS of 2-4<br>• FMMS score of 55 or less	• Adverse Events	• NIHSS<br>• mRS<br>• Fugl-Meyer Motor Score (FMSS)<br>• MMSE<br><br>Additional Outcome Measure:<br>• *Magnetic Resonance Imaging (MRI) analysis<br>• *Positron Emission	1 year & 2012.6 to 2018.5 (Active/ not recruiting)	China

		• Two evaluations at approximately 3 weeks apart prior to surgery with less than +/- 4 point change in the NIHSS<br>• Able and willing to meet all follow-up requirements and undergo post-physical therapy/rehabilitation		Tomography (PET) analysis		
Pilot Investigation of Stem Cells in Stroke Phase II Eficacy (PISCES II) (NCT02117635)	Phase II & NSC (CTX DP)	• Written informed consent or witnessed informed consent (if the patient is unable to sign informed consent due to paresis of the affected arm)<br>• Supratenorial ishaemic stroke<br>• Male or female aged 40 years or more.<br>• Modified NIHSS of 2-4<br>• Clinical diagnosis of stroke confirmed by physician using neuro-imaging<br>• A Score of 0 or 1 for test 2 of the ARAT on day 28+7 and day 56+7 post-stroke using the affected arm.<br>• Ability to comprehend verbal commands.<br>• Eligible for neurosurgery including appropriate anatomical target for cell implantation.	• ARAT	• Action Research Arm Test (ARAT)<br>• NIHSS<br>• Rankin Focused Assessment (RFA) version of the MRS<br>• BI<br>• FMMS	1 year & 2014.4 to 2018.7 (Completed)	United Kingdom
Investigation of Neural Stem Cells in Ischemic Stroke (PISCES III) (NCT03629275)	Phase II & NSC (CTX0E03)	• Ischemic stroke that includes the supratentorial region, occurring within 6 to 24 months of the time that surgical intervention will be performed (Qualifying Stroke Event)<br>• mRS of 3 or 4<br>• Some residual upper limb movement<br>• Sufficient cognitive and language abilities to comprehend verbal commands and to carry out the study assessments<br>• No medical conditions that would preclude neurosurgery	• mRS	• BI<br>• Timed Up and Go Test (TUG)<br>• Chedoke Arm and Hand Activity Inventory (CAHAI)<br>• Symbol Digit Modalities Test	6 month & 2018.8 to 2022.11 (Recruiting)	United State

		• Ability to attend study visits and complete all study assessments		• Controlled Oral Word Association tasks<br>• Multilingual Naming Test<br>• Montreal Cognitive Assessment<br>• NIHSS<br>• FMMS<br>• Stroke Impact Scale (SIS)<br>• EQ-5D		
A Phase II/III Adaptive Crossover Study of Intracerebral Transplantation of Neural Stem Cells for the Treatment of Paralysis Due to Ischemic Stroke (ChiCTR1800014354)	Phase II/III & hNSC NSI-566RSC	• Have the ability to understand the requirements of the study, provide written informed consent and authorization for the use and disclosure of Protected Health Information (PHI)<br>• Men and women 30-65 years old<br>• Women must have a negative serum pregnancy test and practice an acceptable method of contraception or be of non-childbearing potential<br>• At least 4 months but no more than 24 months from time of stroke at the time of screening, with a motor neurological deficit<br>• Documented history of completed ischemic stroke in subcortical region of middle cerebral artery or lenticulostriate artery with or without cortical involvement, with correlated findings by MRI<br>• mRS of 2-4<br>• FMMS score of 55 or less	• Vital signs treatment-emergent adverse events<br>• Clinical laboratory tests<br>• Physical examination<br>• Whole central nervous system (CNS) MRI<br>• FMMS	• NIHSS<br>• mRS<br><br>Additional Outcome Measure:<br>• **Fluorodeoxy-glucose*-positron emission tomography (FDG-PET)<br>• *Diffusion Tensor Imaging (DTI) of Brain<br>• * functional MRI	2018.8 to 2021.6	China

		• Two evaluations at approximately 3 weeks apart prior to surgery with no more than +/- 4 point change in the NIHSS<br>• Able and willing to meet all follow-up requirements and willing to undergo post-physical therapy/rehabilitation.				
A single center randomized controlled trial for human-derived neural stem cell in the treatment of ischemic stroke (ChiCTR1900022741)	hNSC	• Aged 18-80 years old<br>• Patients with ischemic stroke 2 to 24 months before screening, and the stroke was caused by unilateral anterior and/or middle cerebral artery<br>• Patients with hemiplegia, NIHSS scores were higher than 6 and at least one limb movement disorder)<br>• Neurologicaly were stable (variety of NIHSS was smaller than 2)<br>• mRS of 2-4<br>• Minimum infarct diameter of 1 cm on MRI<br>• Informed consent signed by patient or family member and can complete follow-up	• Incidence of adverse events<br>• NIHSS	• mRS<br>• BI<br>• MMSE<br>• Brain MRI	2019.7 to 2021.6	China
